# Unlocking the Power of Benchmarking: Real-World-Time Data Analysis for Enhanced Sarcoma Patient Outcomes

**DOI:** 10.3390/cancers15174395

**Published:** 2023-09-02

**Authors:** Bruno Fuchs, Georg Schelling, Maria Elyes, Gabriela Studer, Beata Bode-Lesniewska, Mario F. Scaglioni, Pietro Giovanoli, Philip Heesen, on behalf of the SwissSarcomaNetwork

**Affiliations:** 1 Sarcoma Service, University Teaching Hospital LUKS, University of Lucerne, 6000 Lucerne, Switzerland; 2 University Hospital USZ, University of Zurich, 8000 Zurich, Switzerland; 3 Sarcoma Service, Kantonsspital Winterthur, 8400 Winterthur, Switzerland; 4 Patho Enge, SSN Reference Sarcoma Pathology, University of Zurich, 8000 Zurich, Switzerland

**Keywords:** IELAS-RWTD/E (interoperable electronic longitudinal absolute structured real-world time data/evidence), MDT/SB (multidisciplinary team/sarcoma board meeting), VBHC (value-based healthcare), AI/ML (artificial intelligence/machine learning), CROMS (clinician-reported outcome measures), PROMS (patient-reported outcome measures), PREMS (patient-experienced outcome measures), IPU (integrated practice unit), SPDT (sarcoma patient digital twin)

## Abstract

**Simple Summary:**

Benchmarking is a crucial tool for healthcare providers to improve quality and efficiency, especially for complex conditions like sarcomas. Sarcomas are a type of cancer that require a multidisciplinary approach to treatment. However, despite adherence to international guidelines, differences in the processes used by these boards can affect patient outcomes and treatment costs. This study compared two multidisciplinary teams/sarcoma tumor boards and established an interoperable digital platform, Sarconnector^®^, for real-world time (RWT) data assessment and automated analysis. Differences were obtained in various areas, such as first-time presentations, follow-up presentations, primary sarcomas, biopsies and chemotherapy indications. By identifying areas of improvement and making data-driven decisions on the meta-level, healthcare providers can optimize resources and improve patient outcomes. Benchmarking with the RWT harmonized data approach provided by the Sarconnector^®^ can help healthcare providers achieve better outcomes for their patients and improve the overall effectiveness of the healthcare system.

**Abstract:**

Benchmarking is crucial for healthcare providers to enhance quality and efficiency, notably for complex conditions like sarcomas. Multidisciplinary teams/sarcoma boards (MDT/SBs) are vital in sarcoma management, but differences in their processes can affect patient outcomes and treatment costs, despite adherence to international guidelines. To address this issue, this study aimed to compare two MDT/SBs and establish an interoperable digital platform, Sarconnector^®^, for real-time-world data assessment and automated analysis. The study included 983 patients, 46.0% of whom female, with a median age of 58 years, and 4.5% of patients presented with metastasis at diagnosis. Differences were observed in the number of first-time presentations, follow-up presentations, primary sarcomas, biopsies and chemotherapy indications between the two MDT/SB. The results highlight the importance of benchmarking and utilizing a harmonized data approach, such as the RWT approach provided by the Sarconnector^®^, to standardize and evaluate quality and cost metrics. By identifying areas of improvement and making data-driven decisions on the meta-level, healthcare providers can optimize resources and improve patient outcomes. In conclusion, benchmarking with the RWT harmonized data approach provided by the Sarconnector^®^ can help healthcare providers improve the overall effectiveness of the healthcare system and achieve better outcomes for their patients in terms of both outcomes and costs.

## 1. Introduction

Surgery is the mainstay treatment in sarcoma care [1,2]. While many standards have been described in sarcoma surgery, such as surgical margins, multidisciplinary approaches, preoperative planning, appropriate surgical techniques with adequate postoperative care and regular follow-ups, and importantly, multidisciplinary team meetings, the overall quality definitions of sarcoma surgery have not been addressed [3,4,5,6,7,8]. In a pivotal landmark paper and based on an international consensus jury approach, Domenghino et al. proposed a framework to evaluate the quality of surgical interventions and to identify areas for improvement, with the potential to improve the assessment of surgical interventions and facilitate the sharing of best practices [9]. The authors highlight the importance of data management, with data-management systems being designed to capture comprehensive and accurate data on surgical outcomes, including clinical outcomes, patient-reported outcomes and complications of therapy. Data need to be interoperable and allow the integration of data from multiple sources, including electronic health records, registries and administrative databases [10,11,12]. Specifically, the consensus jury suggests assessing a multilayer outcome to compare results from one’s own practices, processes, or outcomes to those of other organizations or practices in the same field, both nationally and internationally, to ultimately allow the establishment of a benchmark as a powerful tool that can be used in many surgical disciplines to improve quality and performance and to establish best practices and standards of care.

Benchmarking in surgery or in healthcare in general is considered difficult for a variety of reasons and has only scarcely been reported up to now [9,13,14,15]. However, besides the potential of quality improvement through improvement of clinical practices, it allows the establishment of standards of care, identifying best practices and outliers, while also easing resource allocations and regulatory compliances [13,16]. Domenghino et al. suggest building the benchmark based on outcome parameters assessed at different time points, on the routine assessment of PROMS and PREMS in clinical care, and on the record of individual and global morbidity according to the Clavien–Dindo classification and the Comprehensive Complexity Index [9,17,18,19,20].

In today’s healthcare system, the traditional fee-for-service model creates misaligned incentives where providers incentivize the delivery of a high volume of services rather than focusing on outcome [21,22]. Porter et al. introduced the value-based healthcare (VBHC) principle to better align incentives with outcome, to focus on patient needs, to emphasize outcome over volume, to encourage continuous improvement and to promote transparency and accountability [23,24,25,26]. He defined the value of healthcare as the ratio of quality to cost implying that to increase the value of healthcare, the quality of care delivered to patients has to increase, while also reducing the cost of that care. By measuring outcomes, therefore, areas of improvement are not only identified but also drive quality-improvement initiatives to improve patient outcomes. Therefore, to create a sustainable healthcare system and to realize VBHC, every effort has to be taken to define quality of care and to create opportunities to benchmark it and scale it over the geography [27,28,29,30].

Obviously, the definition of quality in patient outcomes to establish a benchmark as outlined above involves a shear amount of data, which on top has to address data governance, data integration, enabling analytics and data interoperability to share and to encourage collaboration, as well as ethical and legal considerations [11,31,32,33,34,35]. Modern strategies involving AI and ML approaches will revolutionize current approaches [12,33,36,37,38,39]. However, although machine-learning approaches to extract comprehensive data from electronic health records are on the horizon [40], a structured data frame for a given medical condition is necessary, allowing for standardization, interoperability, data analytics, security, transparency, collaboration and further improvements to enable data harmonization over the geography. Above all, real-time follow-up over the entire care cycle, including both clinician and patient perspectives is highly preferable, should be integrated by an interoperable data platform, which ultimately allows federated exchange and learning [41,42,43].

With respect to sarcoma, our group has recently established the spectrum of sarcoma surgery, the complexity scores for the surgery of soft tissue tumors, as well as the quality indicators of sarcoma care [44,45,46]. These consists of six groups, namely the MDT/SB-management, therapy-related parameters including surgery, radiation oncology and chemotherapy, the complexity of sarcoma therapy, physician-based clinical metrics (summarized as CROMS; clinician-reported outcome parameters), as well as patient-based outcome and experience measures (PROMS/PREMS). We have also introduced the sarcoma-specific instrument to longitudinally assess health-related outcomes of the routine care cycle from sarcoma patients’ perspective [47,48]. To realize VBHC [49], it is our strategy to integrate the outlined data complexity by establishing real-world time data exchange, introducing an interoperable platform to benchmark outcome and to align quality with costs.

Therefore, this article addresses the need to define quality indicators and establish benchmarking in sarcoma surgery and care. Our study fills the gap by proposing a framework implemented through an interoperable digital platform. We present a dataset of parameters for benchmarking sarcoma care, enabling the harmonized comparison of multidisciplinary teams and their (surgical) outcomes on the meta-level. This contribution is significant as it facilitates the assessment and improvement of (surgical) interventions, promotes best practices, and establishes standards of care. Additionally, our study aligns with the principles of value-based healthcare, emphasizing patient outcomes, continuous improvement, and cost reduction. By integrating data complexity on the meta-level and introducing an interoperable digital platform, we pave the way for sustainable healthcare systems and improved patient outcomes in sarcoma care.

Herein, we introduce the Sarconnector^®^ (BF&PH, Zurich, Switzerland) as an interoperable digital platform to pave the way for the benchmarking of sarcoma care through real-world time data assessment of automated analysis. We report the dataset of parameters to define the outcome and quality indicators used for benchmarking and present as the proof of principal of assessing meta-level data (as opposed to the more familiar ground-level data) the comparison of two independent MDT/SBs with its automated data analysis.

## 2. Materials and Methods

### 2.1. Study Objectives

The primary objective of this study was to compare the demographics and basic treatment plan of two independent MDT/SBs to set the stage for prospective, large-scale, electronic, structured, longitudinal over the entire care cycle, with consecutive and absolute patient numbers, real-world time data assessment, as well as its automated analysis to create evidence regarding sarcoma care. The second objective was to establish and integrate an interoperable digital platform herein called Sarconnector^®^, which fulfills all the outlined requirements and allows its use in the daily routine work process.

### 2.2. Study Population 

Data from patients diagnosed with sarcoma and presented at two independent MDT/SB sarcoma centers (MDT/SB-A and MDT/SB-B) were consecutively included and prospectively collected over 15 months. They both included one main tertiary referral University hospital each and its associated hospitals and networks. At both of these MDT/SB, more than 100 newly diagnosed patients with sarcomas each year are being discussed, thereby qualifying as internationally representative sarcoma centers [3]. For both MDT/SB, the same interoperable digital platform was used to assess the information of the patients. For both MDT/SB, it is a prerequisite to have a pathology reference review available to review all relevant imaging studies, and to have all 8 disciplines participating at the respective weekly meetings. All newly diagnosed patients, all patients after completion of each treatment step (for example, if combination therapy is decided on, the patient has to be presented after completion of preoperative radiation therapy and before surgery) or patients with a change to the treatment plan other than previously decided on are required to be presented at the MDT/SB.

### 2.3. Sarconnector^®^

**Figure 1:**
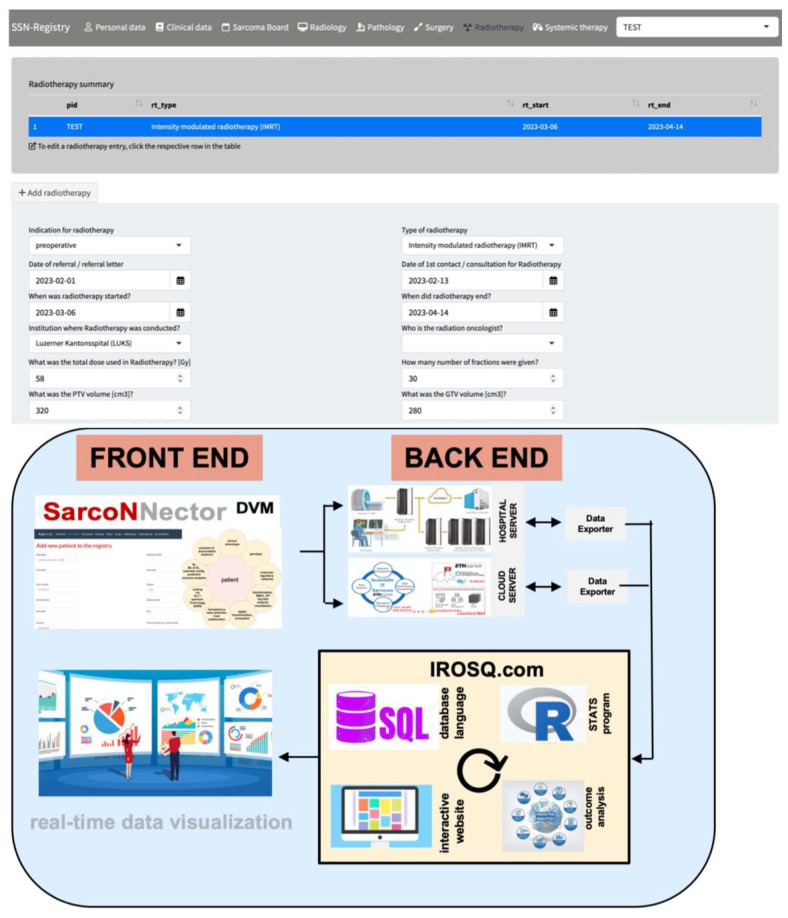
The Sarconnector^®^ as an interoperable digital platform to allow IELAS-RWTD/E. An integrated practice unit (IPU) with an interoperable digital platform is a prerequisite to assessing IELASRWTD/E. A data quality guarantor creates the link between the interoperable digital platform, herein referred to as the Sarconnector^®^, which combines the assessment of data and simultaneous analysis with descriptive, inferential, non-/parameter and Bayesian statistics, with a great focus on exploratory data analysis and visualization. The front end consists of an easy-to-use data entry site, which simultaneously allows visualization of the data. The core of the back end is based on the SQL database language with the R program to perform statistical analysis.

**Figure 2:**
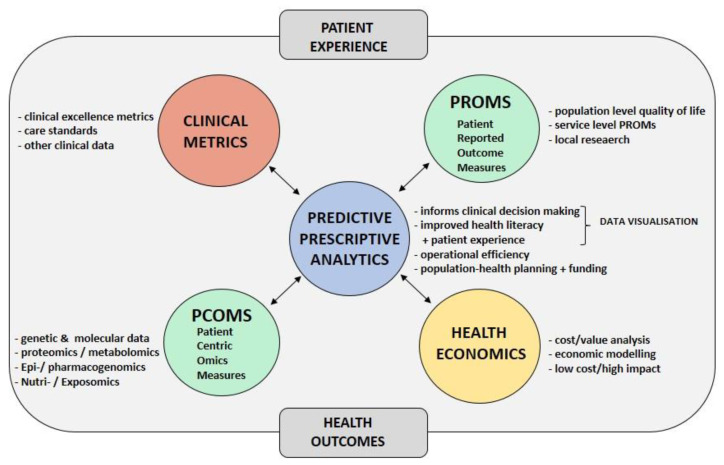
The Sarconnector^®^ includes all data dimensions. This interoperable digital platform (Sarconnector^®^) includes all relevant physician-based, work-up, therapy and follow-up data, as well as patient-based PROMS and PREMS. To associate costs with outcome, it also includes health economics data. Based on these data, AI/ML approaches can be applied for predictive and prescriptive outcome modelling, ultimately enabling the sarcoma patient digital twin.

The interoperable digital platform is introduced elsewhere [46,47]. It is now expanded to the Sarconnector^®^, which presents with a front end as well as a back end (Figure 1). The front end includes both the data entry and the real-time data visualization. The core of the back end bases on the SQL database language with the R program to perform statistical analysis. Data are introduced either through the hospital or cloud server using API data exporter tools and interactive shiny apps. RWTD/E assessment is made possible through the combination of the weekly MDT/SB and the interoperable platform, as well as through PROMS/PREMS assessment by patients during their life-long follow-up [46,47]. Besides PROMS/PREMS, the Sarconnector^®^ also includes clinical metrics (so-called CROMS) and health economics as data dimensions (Figure 2). Because of the RWTD assessment set-up, personalized and automated analytics can also be carried out in real-time.

The Sarconnector^®^ is designed to synthesize meta-level data to integrate multiple MDT/SB. As such, it provides not only object- or ground-level data, but specifically meta-level data, which yields higher-level analysis that is concerned with the structure, organization or properties of a lower level, related to higher-level thinking. It analyzes the quality indicators for a specific MDT/SB separately but can also integrate the data over several MDT/SB, thereby establishing a benchmark not only for a respective institution with its associated network, but also for a country or continent.

The flowchart of data processing by the Sarconnector^®^ (“Driving precision in sarcoma care: real-world time data, value-based benchmarking, and digital twinning”) can be summarized as follows: (A.) Collection of real-world time data over the entire care cycle: data related to sarcoma care, including clinical-reported outcome measures (CROMS), patient-reported outcome measures (PROMS), patient-reported experience measures (PREMS) and quality indicators (QIs), are collected from various sources, such as electronic health records, surveys and patient feedback; (B.) Storage of data on an interoperable digital platform: the collected data are stored securely on a digital platform that allows for interoperability, ensuring compatibility and data exchange between different systems and stakeholders; (C.) Automated analysis on the platform with immediate front end display: the platform employs automated analytical tools and algorithms to process the data and extract relevant insights. This involves statistical analysis, data mining and machine-learning techniques to identify patterns and trends; (D.) Benchmarking and quality indicators: the analyzed data are compared against predefined quality indicators specific to sarcoma care. These indicators include measures such as survival rates, recurrence rates, patient satisfaction scores, adherence to treatment guidelines, etc.; (E.) Assessment of sarcoma care quality: based on the benchmarking results, an assessment can highlight areas of strength and areas that require improvement; (F.) Value-based healthcare assessment: evaluating the value provided by the care process, this assessment takes into account the outcomes achieved relative to the resources used. It considers the effectiveness, efficiency and patient-centeredness of the care provided, aiming to optimize the overall value delivered to patients; (G.) Iterative improvement loop: the assessment findings are used to identify areas for improvement in the sarcoma care process. The healthcare team can take corrective actions, update protocols and implement interventions to enhance the quality of care provided; (H.) Predictive AI/ML modelling: the collected real-world time data are leveraged to develop predictive models using artificial intelligence and machine-learning techniques. These models can forecast future outcomes, identify high-risk patients and support personalized treatment decisions, enhancing the decision-making process; (I.) Composition of the sarcoma patient digital twin: over time, as more data are collected and analyzed, the information is utilized to create a digital twin of sarcoma care. This digital twin serves as a virtual representation that can stimulate the behavior of the care process, predict outcomes and support decision making.

### 2.4. Statistical Analysis

Descriptive statistics were used to describe baseline patient characteristics. Categorical variables are presented as N (%), while numerical variables are presented as median (range). Fisher’s exact test was performed to test for differences in chemotherapy and biopsy proportions between the two MDT-SBs. Statistical analysis was performed using the R statistical program.

## 3. Results

### 3.1. Basic Data and the Sarconnector^®^

Overall, there were 983 patients included in this study, of which 452 (46.0%) were female, with a median age at diagnosis of 58.0 (range, 1.0 to 59.0) years. Table 1 summarizes the dignity as well as the anatomic location of the lesions. There were 44 (4.5%) patients who presented with metastasis at diagnosis.

The Sarconnector^®^ presents an intuitive, self-explanatory front end, separated according to the respective disciplines (Figure 1). For each discipline, a minimal dataset of relevant parameters are requested to enter. The case report form is provided in the supplementary data (SUPP). At the top, it provides a summary of what type of, e.g., radiation therapy was performed. Specifically, besides the type of radiation performed, it also includes the use of a flab, the critical tumor volume/gross tumor volume (CTV/GTV) volumes, and the color wash. Because all disciplines assess their respective information regarding work-up and therapy, these parameters can be analyzed in relation to each other.

### 3.2. Comparison of Two MDT/SB

A basic data benchmark framework of consecutive patients over a 15-month period of two MDT/SBs were assessed for comparison (Table 2). While there were twice as many first-time presentations in MDT/SB-A compared with MDT/SB-B, there were equal follow-up presentations for both MDT/SBs. Patients with primary sarcomas were more numerous in MDT/SB-A than in MDT/SB-B, but together, they totaled 321 patients. An important difference relates to the number of biopsies, which are twice as much in MDT/SB-A as opposed to -B. This might be explained partly by the increased number of benign lesions being presented to the MDT/SB-A. While the indications for surgery and radiation therapy are comparable, the indications for chemotherapy differ between the two MDT/SBs. There are important differences pointing towards different strategies with respect to work-up and therapy. For example, there was a statistically significant difference in the proportion of biopsies performed. In the MDT/SB-A, 523/610 (85.7%) patients received a biopsy, but only 259/373 (69.4%) patients did in MDT/SB-B, *p* < 0.0001. Likewise, 23/330 (6.9%) patients with a sarcoma in MDT/SB-A were treated with chemotherapy, while 83/304 (27.3%) of sarcoma patients in MDT/SB-B were treated with chemotherapy, *p* < 0.0001.

### 3.3. Interactive Data Analysis and Visualization

**Figure 3:**
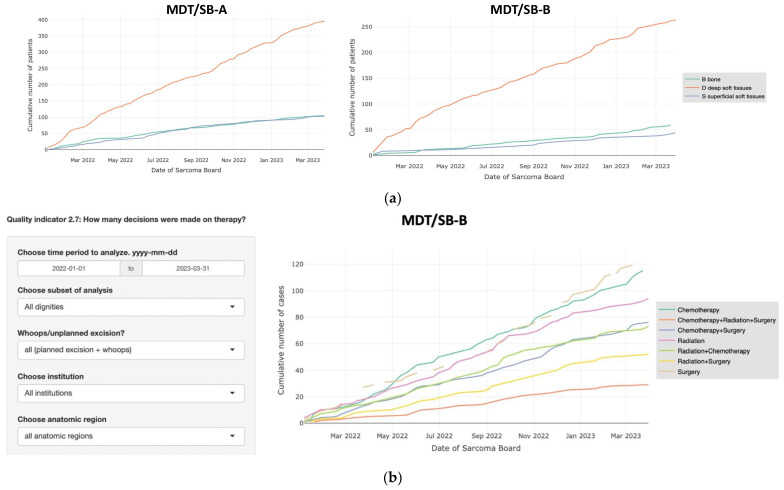

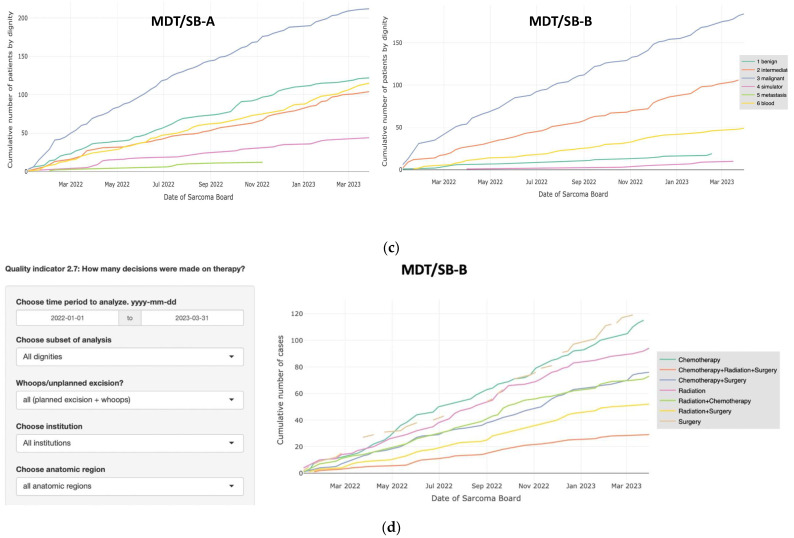
(**a**) These graphs show the cumulative incidence of patients presented at the MDT/SBs over a 15-month period, according to anatomic location of the tumor. (**b**) These graphs show the cumulative incidence of the patients presented at the MDT/SBs over a 15-month period, according to dignity of the tumors. (**c**) This graph shows the cumulative indications of patients presented at the MDT/SB-A over a 15-month period, according to the performed therapy. (**d**) This graph shows the cumulative indications of patients presented at the MDT/SB-Bs over a 15-month period, according to the performed therapy.

The Sarconnector^®^ allows the interactive comparative visualization of the basic data with respect to a given time period, according to anatomic location, tumor biology or dignity of the tumor, and according to therapy, side by side for the respective MDT/SB (Figure 3a–d). The incidence of diagnosis or type of therapy is visualized over time to easily compare subgroups. Visualization of data is critically important to define subgroups for detailed analysis. Importantly, based on the selection of the subgroups to be defined for analysis, the system links the graphs with the respective raw data such that further detailed analysis can be carried out.

### 3.4. Automated Statistical Analysis and Visualization

**Figure 4:**
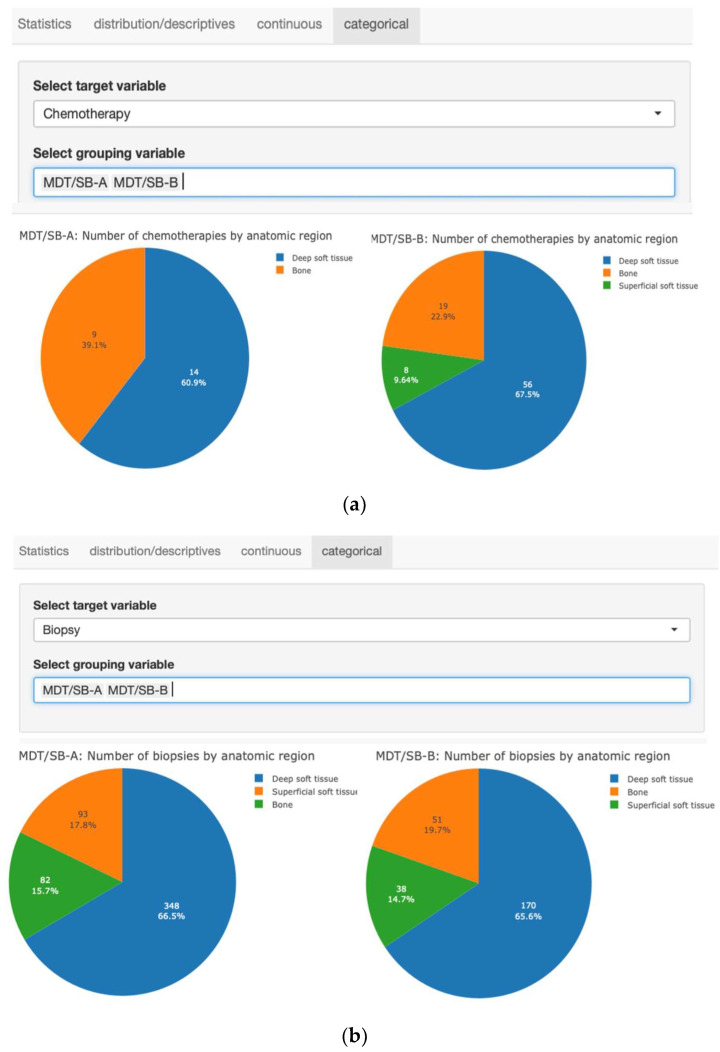
(**a**) There was a statistically significant difference in the proportion of biopsies performed. In the MDT/SB-A, 523/610 (85.7%) patients received a biopsy, but only 259/373 (69.4%) patients did in MDT/SB-B, *p* < 0.0001. (**b**) There was a statistically significant difference in the proportion of chemotherapies performed. In the MDT/SB-A, 23/330 (6.3%) patients with a sarcoma were treated with chemotherapy, and 83/304 (27.3%) patients were in MDT/SB-B, *p* < 0.0001.

The Sarconnector^®^ allows the analysis of any subgroup parameter, the performance of basic statistical tests, as well as advanced statistical techniques (such as Cox regression, competing risk analysis). As a first step of the workflow, the appropriate statistical measure to analyze the data is chosen. For example, a Shapiro–Wilk normality test and Levene’s test for equal variances are performed. Since these tests might suffer from low power [50], the researcher can also visually assess normal distribution by inspecting normal Q-Q plots and histograms. In the next step, the appropriate test is automatically chosen and performed. Furthermore, summary statistics (such as means and standard deviations of the compared samples or Kaplan–Meier estimates) and the corresponding figures are produced, which can be used in scientific publications (such as this one). The Sarconnector^®^ therefore facilitates the conduction of clinical studies (Figure 4).

Figure 4 shows an interactive statistics tool of the Sarconnector^®^, which allows the performance of statistical tests (such as the *t* test) for continuous and categorical variables and the drawing of figures for publications automatically; (Figure 4a.) biopsy and (Figure 4b.) chemotherapy are shown as representative examples.

## 4. Discussion

We herein present a novel approach to handling and harmonizing medical data, thereby mirroring sarcoma patient care in real-world time and comparing respective sarcoma centers/IPUs. Large amounts of data are being assessed trans-disciplinarily and trans-institutionally, as well as across centers using the Sarconnector^®^, which is designed to determine quality indicators of sarcoma care and to provide a quality-management system. It is important to realize that the Sarconnector^®^ does not simply collect data on the object-level but on the meta-level. Herein, we report the comparison of two large sarcoma centers in terms of how patients are being cared for to be subjected for meta-level data analysis. For example, we found important differences regarding the work-up approach of biopsies, as well as of therapeutic approaches, such as the indication to use chemotherapy. This provides unexpected insights about providing sarcoma care of different healthcare ecosystems, with potentially important consequences for both quality and longitudinal cost of care, thereby allowing the establishment of a benchmark. The Sarconnector^®^ with its numerous critical care parameters being harmonized, interoperable and benchmarked over the geography not only allows automated evidence-based insights from the entire care cycle of an individual patient, but also ultimately paves the way for value-based precision care, which may represent the main strength of the novel meta-level approach as presented herein.

Obviously, there are also limitations to consider. The findings reported herein heavily rely on the availability and accuracy of the data collected. Missing or incomplete data could impact the analysis and potentially introduce biases. Also, two large sarcoma centers may not fully represent the diversity of sarcoma care across all healthcare ecosystems. Further, while the Sarconnector^®^ identifies associations between different care approaches, it may not establish causality. Confounding factors that were not accounted for in the analysis could influence the observed differences. And while we are focusing on sarcoma care herein, benchmarking might not cover all aspects of healthcare delivery or other medical conditions. Last but not least, while this approach presented herein is novel and comprehensive, there might be challenges or complexities in the implementation and widespread adoption of such a system in RWT healthcare settings.

In healthcare, benchmarking targets define the standard approach to improve patient outcomes, including the establishment of the best achievable real-world postoperative outcomes [15]. Ideally, defined parameters are reproducible, objective and universal [51]. The purpose of benchmarking is to stimulate the genuine endeavor for perfection, rather than judge a unit or physician performance [13,15,52,53]. An international jury consensus approach identified benchmarking as one of the key elements to reporting and improving the quality of surgical interventions and medical care [9]. To realize benchmarking, CROMS, PROMS/PREMS and complications of (surgical) treatments as outcome parameters have to be assessed both nationally and internationally. Why do we need benchmarking? By comparing their own performances with peers, best practices can be identified and learning experiences maximized. The best outcome can be defined and be adopted for standard practices. By comparing clinical practices and outcomes, not only can best practices be identified, but so can new and innovative ways to improve patient care. Benchmarking helps recognize outliers performing exceptionally well or poorly, and subsequently then helps investigate the reasons for their performance. By identifying areas where resources can be best utilized, resource allocation is handled more efficiently. As presented herein, biopsies or chemotherapies may be performed too often or not, but having the opportunity to align with outcome will enable the system to optimally allocate resources. In the context of accreditation and certification programs, healthcare providers comply with regulatory requirements and demonstrate the quality of care provided. Benchmarking in medical care and specifically cancer care, however, is not widespread. Furthermore, it has to be distinguished from standard of care, which generally refers to accepted practices, protocols and guidelines for providing medical treatment for a particular condition or disease [1,2]. Such standards are established based on the best available scientific evidence and expert consensus and are often used as a benchmark or reference point for evaluating the quality of care. In contrast, benchmarking involves comparing the performance of healthcare providers or healthcare systems against established standards or against each other. The standard of medical care provides a set of guidelines and expectations for how medical care should be delivered, while benchmarking medical care involves comparing actual performance against those standards to identify areas of improvement and drive quality improvement. In both herein presented MDT/SBs, the provided care is based on guidelines; nevertheless, important differences in practicing do exist, emphasizing the importance of introducing a benchmark tool in addition to the established guidelines. While there are no benchmarks reported for sarcoma care, there are sparse studies in the literature on cancer care and some on surgical disciplines. The international cancer benchmarking partnership (ICBP) is a collaboration of researchers and clinicians from several countries (Australia, Canada, Denmark, Ireland, New Zealand, Norway, Sweden and the United Kingdom) that aims to investigate and explain differences in cancer survival between countries [54,55]. This partnership compares cancer survival rates and stage at diagnosis across different countries using a standardized methodology in order to identify factors that contribute to variations in cancer outcomes [55]. The ICBP’s mission statement is to provide policymakers, health professionals and the public with information about international variations in cancer survival and factors that might contribute to it in order to improve outcomes. To achieve this mission, the ICBP conducts research and analysis on cancer survival rates, stage at diagnosis and other related factors across participating countries. While this approach is laudable, it only concentrates on specific aspects of sarcoma care, while the herein presented approach is holistic. Nolte et al., exploring the link between policies and cancer survival, found a positive correlation with improvements in survival over time across various cancer sites analyzed [53]. Perera et al. developed an evidence-based benchmark rate for cancer surgery used to provide a new template for high-income and emerging economies to rationally plan and assess their cancer surgery provisions [56]. Wind et al. explored the possibilities to benchmark cancer centers by structuring cancer care into pathways, reducing variability in clinical practices and improving patient outcomes [16]. They were successfully establishing and testing a benchmark tool that was pivotal to organizing cancer care in an IPU (integrated practice unit) to yield multiple performance improvements [52]. While benchmarking approaches are sparse for the musculoskeletal system [57], they are fairly common in visceral surgeries [58,59,60,61,62]. While these benchmarks focus on specific one-dimensional diseases, and while it is clear that policies will need to guide the transformation process, there are now efforts being undertaken to establish benchmarks for bottom-up use in more complex diseases [9,13,14]. With respect to sarcomas, there is only little information about benchmarking available [63,64,65], and they all refer to specific entities or surgeries without a holistic bottom-up approach including all basic parameters, underlining the necessity of our bottom-up approach as presented herein. Conversely, if it is the shared goal to benchmark quality and outcome, then it only makes sense to design a system including basic parameters that is designed to do so, i.e., to allow data harmonization and their scaling [44,45,46,47,48]. As shown herein (considering the discrepancies of performing biopsies or the indication to treat with chemotherapy among MDT/SBs and thereby identifying potential areas of differences in quality and costs), designing a system that allows not only the outcome but also the granular pathway of decisions, such as during an MDT/SB, is of critical importance. The Sarconnector^®^ is designed to assess quality indicators, covering all the respective index and outcome parameters, and analyzes the pathway of decisions during MDT/SB to reproduce why which treatment was initiated. Taking into account the inclusion of long-term follow-up to incorporate outcome discrepancies, such an interoperable digital platform has the potential to ultimately become a sarcoma patient digital twin (SPDT) [40,66].

Data structures with their different types are obtained increased focus, and there is continued debate about its use [39,67]. Electronic health records (EHRs) are routinely used for clinical care and research. Clinical trials databases (CTDs) contain collected data during randomized trials. Administrative claims data representing billing codes and information submitted to insurance companies for reimbursement are usually again stored separately. There are also biobanks that store biological samples and associated data. Each data structure has its strengths and limitations [68]. The choice of data structure depends on the research question and the resources available for data collection and analysis. The advantage of real-world data includes the potential to capture real-world complexity, such as contextual factors and system interactions, which may be difficult to simulate through modelling alone [31,32]. Further, RWTD provides a rich source of information that can be used to calibrate and validate models [69,70]. RCTs are considered the gold standard for medical research as they involve randomization. Because these are typically conducted under highly controlled conditions, they can lead to limitations in terms of generalizability to the broader population and are usually associated with great costs. Conversely, RWTD provides a more comprehensive view of healthcare outcomes as it includes data collected from routine medical practice, electronic health records, administrative claims, and patient-generated data [40,67,69,71]. If incomplete or inaccurate data, confounding factors and selection biases are addressed with respective data structures, RWTD has the potential to complement and enhance the insights gained from RCTs [69,71,72,73]. An interoperable digital platform helps to overcome the limits by integrating data from various sources and standardizing data formats [74,75]. With an interoperable platform, data can be accessed, shared and analyzed more easily and efficiently. This may facilitate the identification of patterns and associations in the data that may not have been apparent otherwise. It improves the accuracy and completeness of data by reducing errors associated with data entry and enabling real-time capture [67]. If data are collected over time as we reported earlier [47], more accurate and timely information becomes available. Interestingly, an interoperable digital platform enables more extensive data analytics and modelling to support decision-making processes, such as predictive modelling and machine-learning algorithms [39,74,75,76]. Integrating all common data sources and dimensions, coupled with the opportunity to analyze it concomitantly, enables the realization of real-world time evidence and prediction [77,78,79], and ultimately, the sarcoma patient digital twin (SPDT) [40,66].

The definition of outcome benchmarks with specific reference to quality indicators for a given disease, as well as an interoperable digital platform that includes economic data dimensions like the Sarconnector^®^ does, represents the prerequisite for value-based healthcare, which aims to optimize the balance between health outcomes and costs [24,26,30,46]. VBHC incentivizes healthcare providers to deliver high-value care by linking reimbursement to quality indicators and promoting the use of shared decision-making tools that take into account patient preferences and values. In this context, the availability of RWTD/E with its associated analysis as presented herein plays a pivotal role. Because the Sarconnector^®^ enables benchmarking sarcoma care and consequently the creation of a sustainable healthcare system, it is pivotal to support VBHC. Measuring and comparing performances across different healthcare providers or organizations becomes possible, while enabling the identification of best practices and areas for improvement in healthcare delivery is also made possible over time, which may lead to better outcomes and increased efficiency [21,80]. Variations in care delivery and outcomes may allow potential areas for cost savings and improved efficiency to be identified. Such tracking of performance over time allows for the evaluation of the effectiveness of interventions and the identification of trends. Health equity can be promoted by identifying and addressing disparities in healthcare. Benchmarking may also support VBHC by setting goals and targets for improving healthcare outcomes and reductions in costs, which can help to align incentives and motivate providers to improve performance [13,26,51,56]. High-performing providers may be used to inform patient decision making and help drive competition and innovation in the healthcare industry. The development of novel payment models makes it possible to incentivize high-value care, rewarding providers for delivering high quality at lower costs. It not only facilitates collaboration and knowledge sharing among healthcare providers and organizations, but it also supports the development of policies and regulations that promote the delivery of high-value care and encourage sustainability of the healthcare system. As presented herein by comparing two MDT/SBs and assessing consecutive and prospective RWT data, we found important differences regarding the number of biopsies and the number of chemotherapies performed for a comparable patient cohort. Both MDT/SBs treat their patients according to their best knowledge and available evidence. Nevertheless, the reported differences imply that depending on which sarcoma center/IPU a patient is being treated in, a patient may be over- or undertreated, or the set-up to deliver care may be differently organized. Independent of the situation, it is obvious that such differences have a direct impact on the financial burden. It also becomes obvious that the number per se of performed chemotherapies, for example, is merely a measure of volume but is not correlated with quality of care, which conversely can neither be associated with costs. The Sarconnector^®^ is capable of identifying differences in delivering care between MDT/SBs from the same country and of even neighboring counties with partly overlapping patient populations. There are likely obvious differences in delivering care at different outcome qualities, but this is for certain at different costs. This is a somewhat unexpected finding, but it evidences the validity and the necessity of such a tool. Obviously, many more questions arise with these findings that need to be answered. We therefore believe that the Sarconnector^®^ represents a powerful tool to develop a sustainable healthcare system.

## 5. Conclusions

In conclusion, benchmarking is a crucial tool for improving healthcare quality and efficiency with respective cost containment. The RWT data approach provided by the Sarconnector^®^ offers a valuable method for evaluating quality and cost metrics in a standardized way, allowing for transparent comparisons between different healthcare providers. This approach enables healthcare providers to identify areas for improvement and make data-driven decisions to optimize their resources and improve outcomes. By utilizing benchmarking and the RWT harmonized data approach, healthcare providers can move towards a value-based care model, where high-quality care is delivered at a reasonable cost. Ultimately, benchmarking with the RWT harmonized data approach provided by the Sarconnector^®^ can help healthcare providers achieve better outcomes for their patients and improve the overall effectiveness of the healthcare system with respect to both outcomes and costs.

## Figures and Tables

**Table 1:** This table summarizes all relevant basic demographic data of the patients included in this study. The numbers are separately listed for each MDT/SB, as well as overall.

	Overall N = 983	MDT/SB-A N = 610	MDT/SB-B N = 373	*p*-Value
Female	452 (46.0%)	283 (46.4%)	169 (45.3%)	0.74
Age at diagnosis	58.0 (1.0, 95.0)	60.0 (8.0, 93.0)	56.0 (1.0, 95.0)	0.001
Bone tumors Chondrogenic Osteogenic Vascular Others/Unknown Soft-tissue tumors Adipocytic (Myo-)fibroblastic Fibrohistiocytic Muscle tumors Undifferentiated/un- classified Others	44 (4.5%) 19 (1.9%) 18 (1.8%) 81 (8.2%) 201 (20.5%) 117 (11.9%) 33 (3.4%) 82 (8.3%) 87 (8.9%) 301 (30.6%)	24 (3.9%) 6 (1.0%) 14 (2.3%) 60 (9.8%) 141 (23.1%) 59 (9.7%) 11 (1.8%) 51 (8.4%) 50 (8.2%) 194 (31.8%)	20 (5.4%) 13 (3.5%) 4 (1.1%) 21 (5.6%) 60 (16.1%) 58 (15.6%) 22 (5.9%) 31 (8.3%) 37 (9.9%) 107 (28.6%)	<0.001
Primary tumor site Appendicular Axial NA	558 (56.8%) 367 (37.3%) 58 (5.9%)	352 (57.7%) 220 (36.1%) 38 (6.1%)	206 (55.2%) 147 (39.4%) 20 (5.4%)	0.37
Metastasis at diagnosis	44 (4.5%)	26 (4.3%)	18 (4.8%)	0.75

**Table 2:** This table summarizes all relevant oncological data of the patients included in this study. The numbers are separately listed for each MDT/SB as well as overall.

	OVERALL	MDT-SB/A	MDT-SB/B	*p*-Value
Total number of patients	983	610	373	<0.001
Total number of presentations	1556	914	642	<0.001
	OVERALL	MDT-SB/A	MDT-SB/B	*p*-value
1st time presentations	650	416	234	<0.001
Follow-up presentations	833	431	402	<0.001
Dignity: 1st time/fup presentation Benign Intermediate Malignant Simulator Metastasis Blood Others	650/833 120/70 135/50 186/548 43/20 10/4 53/11 103/30	416/431 105/54 61/77 99/256 35/13 10/4 9/1 97/26	234/402 15/16 74/3 97/292 8/7 0/0 44/10 6/4	<0.001/0.17 <0.001/<0.001 <0.001/<0.93 <0.001/<0.001 0.01/0.26 0.02/0.13 <0.01/0.005 <0.01/<0.01
Localization: 1st time/fup presentation Bone Deep soft tissues Superficial soft tissues NA	650/833 117/141 431/579 95/103 7/10	416/431 77/76 269/290 70/62 0/3	234/402 40/65 162/289 28/41 4/7	<0.001/0.17 0.67/0.58 0.26/0.15 0.11/0.07 0.02/0.21
1st time & intermediate/malignant Bone Deep soft tissues Superficial soft tissues	321 48 224 49	160 20 111 29	161 28 113 20	0.99 0.27 0.90 0.17
Total number of biopsies Bone Deep soft tissues Superficial soft tissues	782 133 518 131	523 82 348 93	259 51 170 38	<0.001 0.19 0.81 0.31
Indications for surgery Bone Deep soft tissues Superficial soft tissues	393 70 259 64	244 38 161 45	149 32 98 19	0.77 0.17 0.99 0.16
Indications for radiotherapy Bone Deep soft tissues Superficial soft tissues	98 5 73 20	46 2 33 11	52 3 40 9	0.48 0.99 0.65 0.46
Indications for chemotherapy Bone Deep soft tissues Superficial soft tissues	106 28 70 8	23 9 14 0	83 19 56 8	<0.001 0.18 0.62 0.20

## Data Availability

Not applicable.
